# Ameliorative effect of *Sedum sarmentosum* Bunge extract on Tilapia fatty liver via the PPAR and P53 signaling pathway

**DOI:** 10.1038/s41598-018-26084-2

**Published:** 2018-05-31

**Authors:** Lida Huang, Yuan Cheng, Kai Huang, Yu Zhou, Yanqun Ma, Mengci Zhang

**Affiliations:** 1 College of Animal Science and Technology of Guangxi University, Nanning, China; 2 Zhanjiang Haiyuan Biological Technology Co. Ltd, Zhanjiang, China; 3 Guangxi Academy of Fishery Sciences, Nanning, China

## Abstract

Fatty liver disease is a growing problem in fish aquaculture and there is an urgent need to identify causes and possible remedies. In the present study, the effects of treating fatty liver disease in the Nile tilapia (*Oreochromis niloticus* Linnaeus, 1758) with an extract derived from a herb, *Sedum sarmentosum* Bunge (SSB), was investigated. We found that the SSB extract could restore the changes to feed coefficient, immune capacity, and pathological index caused by fatty liver disease, and also prevent apoptosis in hepatocytes. An RNA-seq analysis showed that treatment with SSB extract altered expression of genes in the lipid metabolic process, metabolic process, and oxidation-reduction process. Our results suggest that disorders of the PPAR and p53 signaling pathways may be involved in steatohepatitis development and in the therapeutic mechanism of the SSB extract treatment; these observations shed new light on possible treatment of steatohepatitis.

## Introduction

A high-fat diet is a current trend in aquaculture due to the increasing cost of protein ingredients and the limited supply of fish meal worldwide. However, a high-lipid diet can increase lipid deposition and lead to a significant accumulation of fatty acids in the liver of fish, which may induce fatty liver disease^1,2^. Currently, fatty liver disease has been reported in aquacultures of a wide range of fish species, including tilapia, grass-fish, rainbow trout, and medaka^3^. The increasing incidence of fatty liver disease in fish cultures has stimulated many investigations into the underlying mechanisms of the disease and into possible means to alleviate this problem.

The causes of fatty liver degeneration are complex; an important factor seems to be an imbalance in the nutrients in daily feed and the lack of particular lipids^4^. For example, in yellow catfish, changes to lipid metabolism lead to lipid deposition in the liver and muscle^5^. Reduction of lipid deposition, increase of metabolites associated with β-oxidation, and reduction of fatty acid levels in the liver, have been shown to improve the fatty liver condition. In the medaka model of fatty liver disease, administration of L-carnitine and eicosapentaenoic acid changed the composition of fatty acids and improved liver condition^3^. Moreover, non-alcoholic steatohepatitis in medaka can be ameliorated by administration of the drug telmisartan, which reduces the infiltration of macrophages into the liver and improves the disease pathology, although the fatty acid content remains unchanged^6^. However, treatment of non-alcoholic fatty liver disease in fish in aquaculture still needs further investigation.

*Sedum sarmentosum* Bunge (SSB) is a perennial herb distributed on mountain slopes in Asia, Europe, and North America^7^. Wei *et al*. isolated a number of active bioactive compounds from this species, including tricin-7-O-β-D-glucoside I, luteolin II, isorhamnetin III, isoliquiritigenin IV, quercetin V, dioctadecyl sulfide VI, and palmic acid VII^8^. The plant has traditionally been employed as a hepatoprotective medicine and for treatment of chronic viral hepatitis^9^. Yoshikawa *et al*. showed that megastigmane glycosides isolated from SSB have a protective effect against cytotoxicity induced by D-(+)-galactosamine hydrochloride (D-GalN) in primary cultured mouse hepatocytes^10^. Moreover, SSB extract can suppress D-GalN/lipopolysaccharide–induced fulminant hepatic failure in mice through its anti-apoptotic activity and inhibition of mitogen activated protein kinase activity^11^. Kang *et al*. reported that SSB extract might improve survival of hepatoma patients by inhibiting the growth of hepatoma cells^7^. It has also been found that SSB extract can inhibit NADPH/vitamin C-induced lipid peroxidation and can protect against hepatocyte injury in rats^12^. To date, the effects of SSB extract treatments have mainly been studied in rodents and little is known about their effects in fish. On the basis of the data from the rodent studies, we hypothesized that treatment with an SSB extract might also have an ameliorative effect on non-alcoholic fatty liver in fish.

Nile tilapia (*Oreochromis niloticus* Linnaeus, 1758) is an important aquaculture species worldwide and is a good fish model for metabolic studies because of its rapid growth, and high disease and stress resistance. In this study, we investigated the effects of SSB extract on non-alcoholic fatty liver disease in tilapia and used RNA-seq to analyze the possible mechanisms of its effects.

## Results

### Diet-induced fatty liver and effect of SSB extract treatment

**Figure 1:**
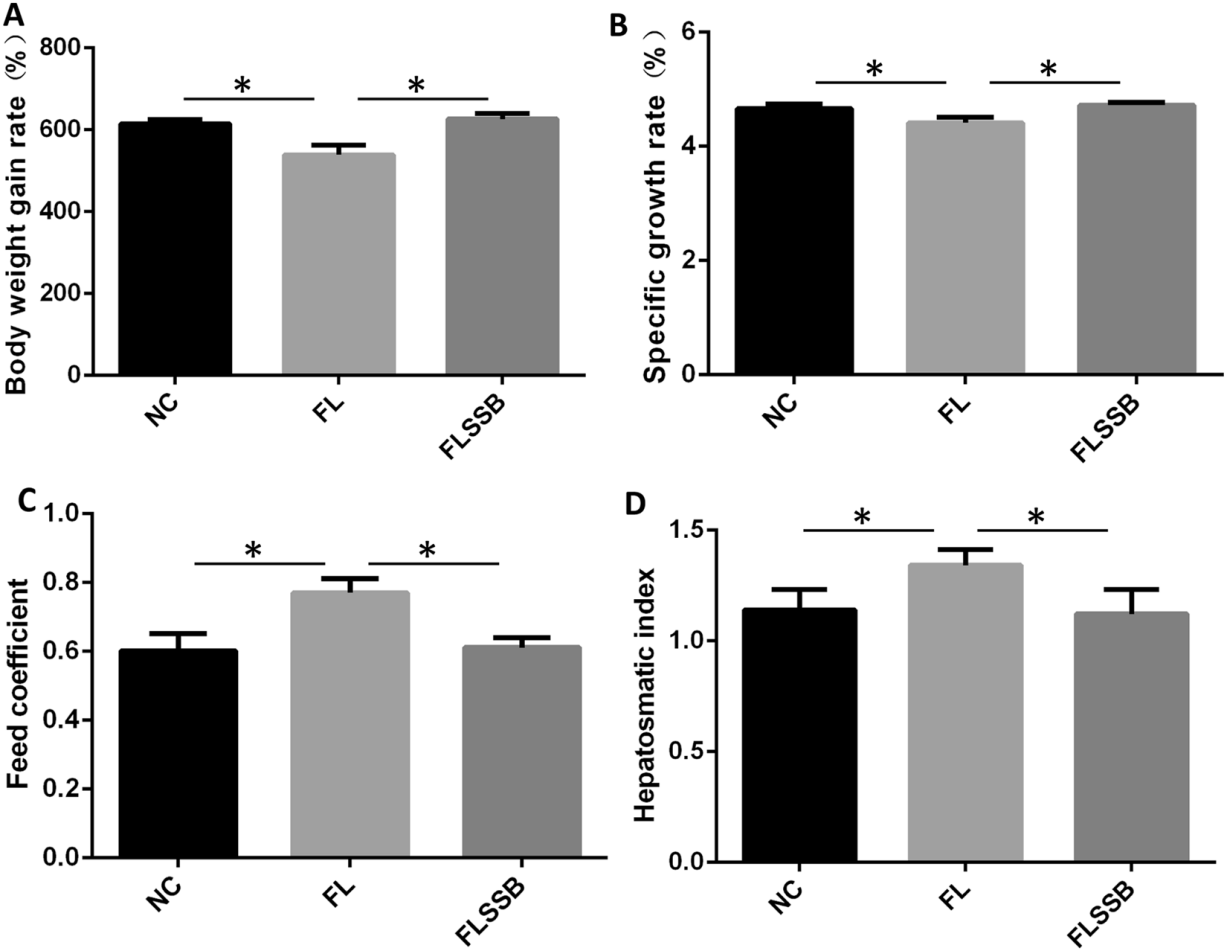
The growth performance of tilapia after 6 weeks of treatment. (**A**) Weight gain rate of tilapia in each group. (**B**) Specific growth rate of tilapia in each group. (**C**) Feed coefficient of tilapia. (**D**) Hepatosomatic index of tilapia in each group. *P < 0.05, (ANOVA, n = 15).

The growth performance of the tilapia was assessed at the end of the 6 week treatment. Weight gain and specific growth rate were significantly lower in the FL group compared to the NC group; by contrast, the FLSSB group showed a significantly increased weight gain and specific growth rate compared to the FL group (Fig. 1A,B). The feed coefficient and hepatosomatic index were higher in the FL group than in the NC and FLSSB groups; there were no significant differences in growth performance between the NC and FLSSB groups (Fig. 1C,D).

### Effect of SSB extract on serum parameters

Analysis of the serum from fish in all three treatment groups showed a higher level of key enzymes for lipid metabolism and absorption, such as TG, in the FL group. Moreover, the liver and kidney functions in the FL group appeared to be seriously damaged as evidenced by the significant increase in AST, ALT, and LDH activities and the levels of CHOL, TBA, and TG (Table 1), compared to the NC group. Notably, treatment with SSB extract inhibited fatty liver formation and restored liver-kidney function in the FLSSB group compared to the FL group. Biochemical markers for liver function, including TP, ALB, GLOB, and ALP, were significantly decreased in the FL group compared to the NC group (Table 2). Treatment with the SSB extract enhanced the levels of TP, ALB, GLOB, and ALP despite the high-fat diet. There were no significant differences between the levels of the biochemical markers in the FLSSB and NC groups.

**Table 1:** Effects of *Sedum sarmentosum* Bunge on blood serum metabolites of Tilapia

Parameters	Groups		
	NC	FL	FLSSB
AST (U/L)	36.00 ± 3.46^b^	72.67 ± 9.07^a^	40.67 ± 4.93^b^
ALT (U/L)	24.00 ± 3.61^b^	56.33 ± 3.97^a^	29.67 ± 3.21^b^
CHOL (mmol/L)	2.66 ± 0.25^b^	3.72 ± 0.06^a^	2.71 ± 0.25^b^
TG (mmol/L)	0.38 ± 0.03^b^	0.56 ± 0.07^a^	0.39 ± 0.05^b^
LDH (U/L)	126.33 ± 5.13b^b^	417.67 ± 25.50^a^	209.33 ± 22.03^b^
TBA (umol/L)	6.30 ± 1.04^b^	17.70 ± 1.85^a^	6.83 ± 1.12^b^

a,b: Values with different superscripts in the same line are significantly different (P < 0.05, ANOVA).

**Table 2:** Effects of *Sedum sarmentosum* Bunge on Immune index of Tilapia

Parameters	Groups		
	NC	FL	FLSSB
TP (g/L)	24.83 ± 0.55^b^	13.67 ± 1.72^a^	24.17 ± 1.46^b^
ALB (g/L)	8.63 ± 0.21^b^	5.87 ± 0.25^a^	8.30 ± 0.79^b^
GLOB (g/L)	16.53 ± 0.23^b^	11.47 ± 1.05^a^	15.87 ± 0.91^b^
ALP (U/L)	22.33 ± 1.53^b^	14.67 ± 1.15^a^	21.67 ± 2.08^b^

a,b: Values with different superscripts in the same line are significantly different (P < 0.05, ANOVA).

### Effect of SSB extract on antioxidant levels

Activities of CAT, SOD, GSH-Px, and T-AOC were significantly lower in the FL group than in the NC group (Table S2). Treatment with SSB extract resulted in a significant increase in CAT, SOD, GSH-Px, and T-AOC activities in the FLSSB groups compared to the FL group, although these levels were still lower than in the NC group. Additionally, the serum levels of MDA were significantly higher in the FL group than in the NC or FLSSB groups; no significant difference was observed between the FLSSB and NC groups. Our observations indicated that development of fatty liver disease was associated with a suppression of antioxidant activity, and that treatment with the SSB extract could restore antioxidant activity.

### Effect of SSB extract treatment on hepatopancreas and digestion index

The activities of AST and ALT in the hepatopancreas of the FL group were higher than those of the NC group, indicating that high-fat diet seriously damaged liver and kidney functions and induced fatty liver disease (Table S3). Notably, treatment with the SSB extract restored liver function as evidenced by the decreased activities of AST and ALT in the FLSSB group compared to the FL group. The level of MDA was much higher in the hepatopancreas of the FL group compared to the NC and FLSSB groups, suggesting that treatment with the SSB extract improved the antioxidant ability of the FLSSB group. The CAT, SOD, GSH-Px, and T-AOC activities of the hepatopancreas of the FL group were significantly lower than those in the NC group or FLSSB group. A digestive enzyme analysis showed that the high fat diet significantly enhanced the activities of hepatopancreatic protease, intestinal protease, hepatopancreatic lipase, intestinal lipase, hepatopancreatic amylase and intestinal amylase. Treatment with the SSB extract reduced the activities of all these digestive enzymes (Table S4).

### Effect of SSB extract treatment on liver pathology

**Figure 2:**
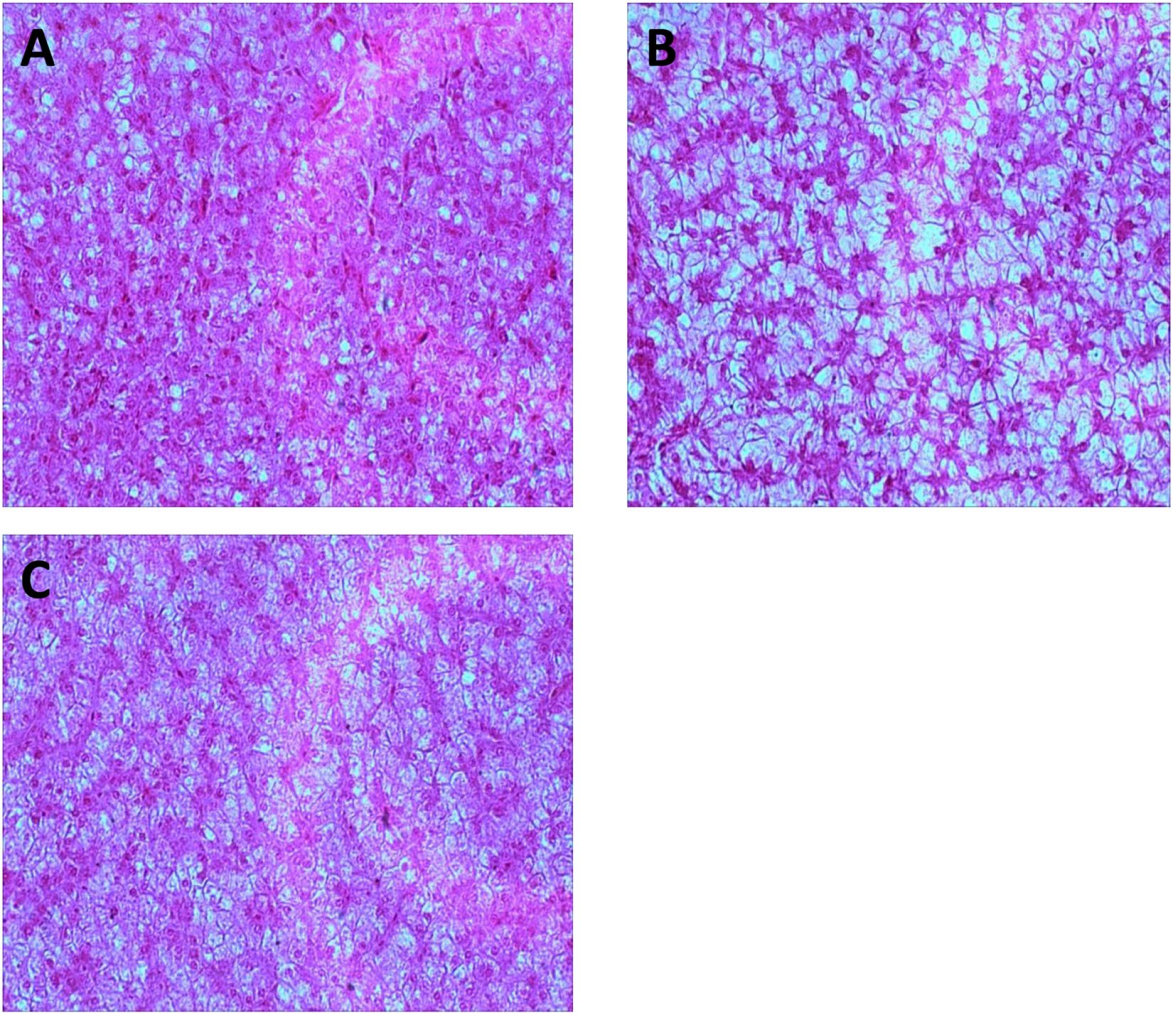
Tissue morphology in the NC, FL, and FLSSB groups. (**A**) NC group. (**B**) FL group. (**C**) FLSSB group.

Analysis of H&E stained liver sections from NC group fish showed the presence of clear cell boundaries, a regular arrangement of hepatic cords, and a lack of cavitations or pathological changes (Fig. 2A). However, in livers from the FL group, 65% of the cytoplasm showed cavitation, and the cells were clearly swollen (Fig. 2B). Moreover, vacuolar lipid droplets were present in the cytoplasm of hepatocytes, and nuclei were displaced, suggesting the establishment of fatty liver disease. By contrast, after treatment with SSB extract, the volume, quantity, and area (38%) of cavitation was reduced, and the degree of swelling of cells was diminished (Fig. 2C). As described in the “Materials and Methods,” we determined the nonalcoholic fatty liver disease activity score. Pathological degeneration, extent of cavitation, and morphology of hepatic cells in the FL group was assessed as grade 3; those of the FLSSB group were assessed as grade 2; and the NC group was assessed as grade 0 (Tables S5 and S6).

### Effect of SSB extract treatment on apoptosis

**Figure 3:**
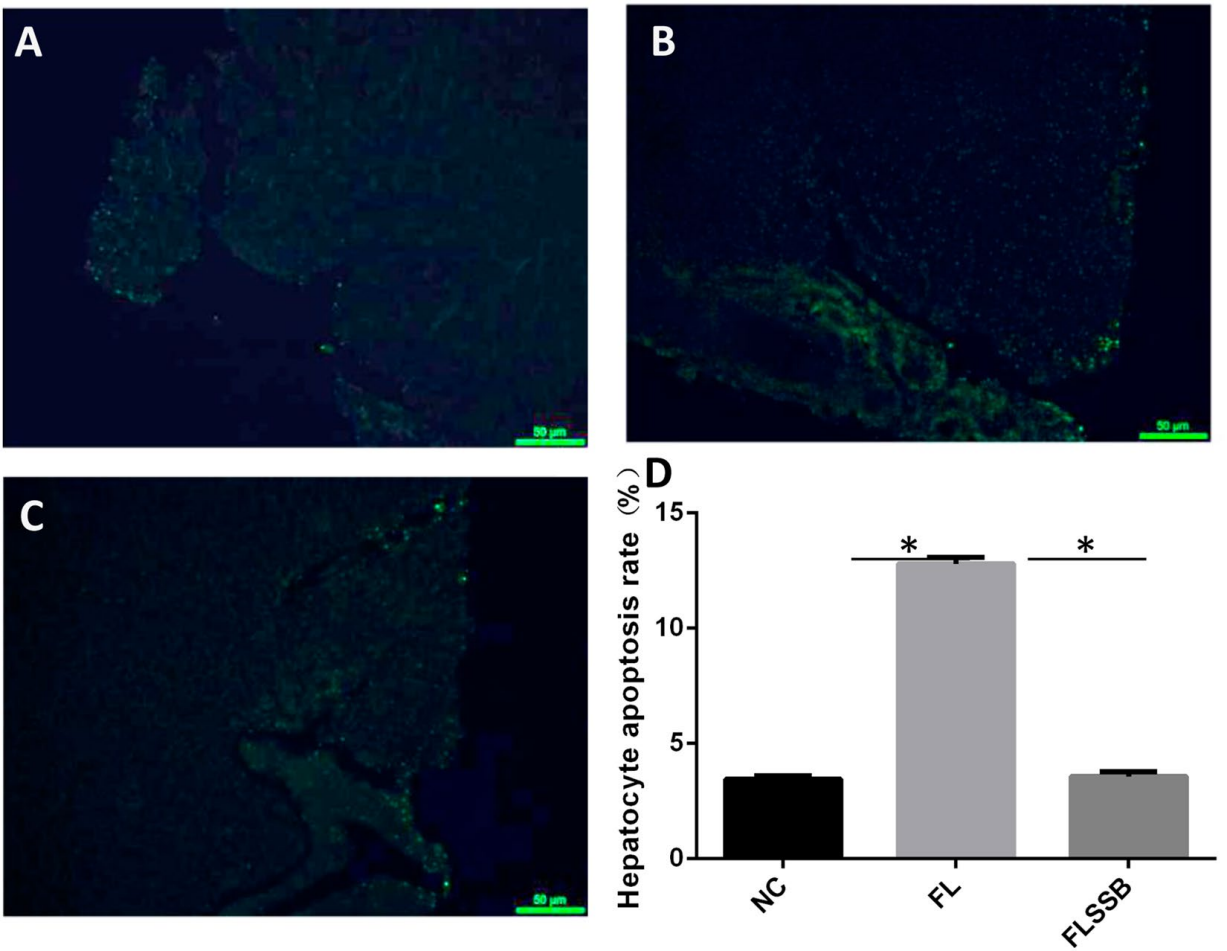
SSB extract treatment decreases hepatocyte apoptosis in liver tissues of tilapia. Hepatocyte apoptosis in the NC group (**A**), the FL group (**B**), and the FLSSB group (**C**). *P < 0.05, (ANOVA, n = 15).

Cell apoptosis in the liver was assayed by TUNEL staining (Fig. 3). A few TUNEL-positive cells were observed in NC and FLSSB groups (<5.0%). More TUNEL-positive cells were present in livers of the FL group. Our observations suggest that treatment with the SSB extract might have inhibited development of fatty liver disease by suppressing liver cell apoptosis.

### Summary statistics for the RNA-seq data

**Figure 4:**
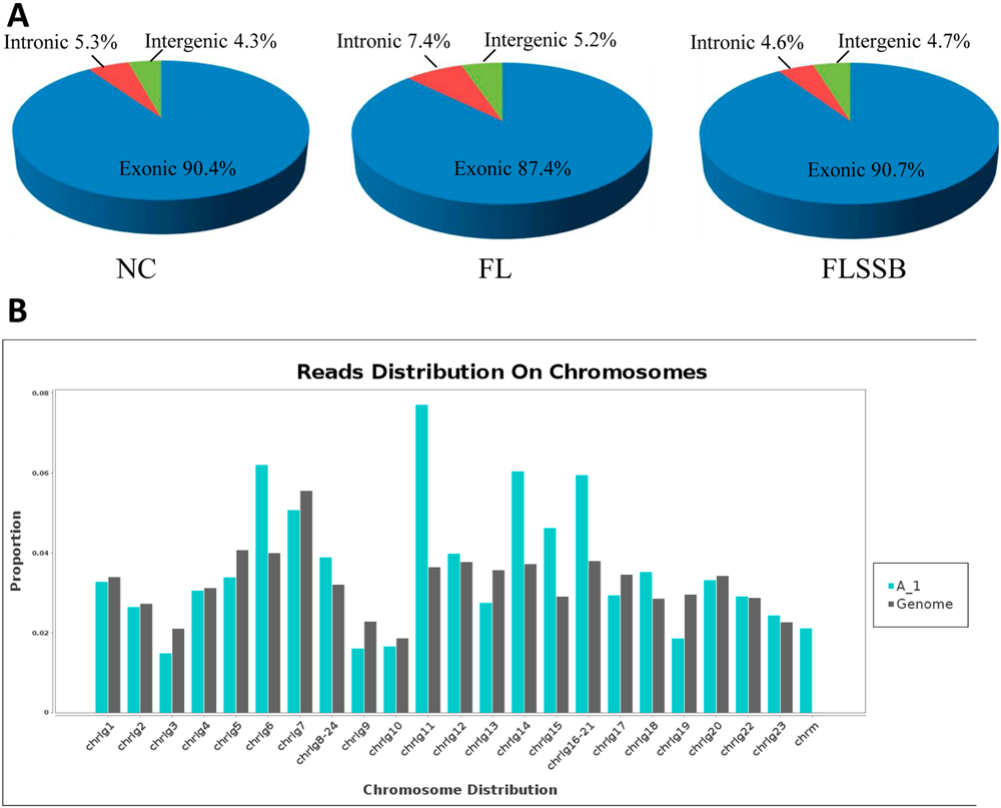
Transcriptomic data from tilapia. (**A**) The proportion of reads mapped to intronic, exonic, and intergenic regions. (**B**) The proportion of reads mapped to different chromosomes.

For each transcriptome sequencing of the 9 liver samples, more than 34 million raw reads were obtained; after cleaning, 72.53% (NC), 72.62% (FL), and 73.20% (FLSSB) reads remained (Supplementary Table S7). Approximately 24 million reads (84.09%) could be aligned to the reference genome, with an average of 22 million reads (79.13%) mapping to unique locations in the *Oreochromis niloticus* genome; a mean of 4 million reads (15.91%) per sample did not map to any location of the reference genome (Supplementary Table S8). Interestingly, the proportion of reads mapping to exonic regions was markedly lower in the FL samples (87.4%) compared to the NC (90.4%) and FLSSB samples (90.7%). Conversely, the proportion of clean reads aligned to intronic regions was higher in the FL samples (7.4%) than in the NC group (4.6%; Fig. 4A). The reads mainly mapped to chromosomes 6, 11, and 14 (Fig. 4B).

### Analysis of differential gene expression

**Figure 5:**
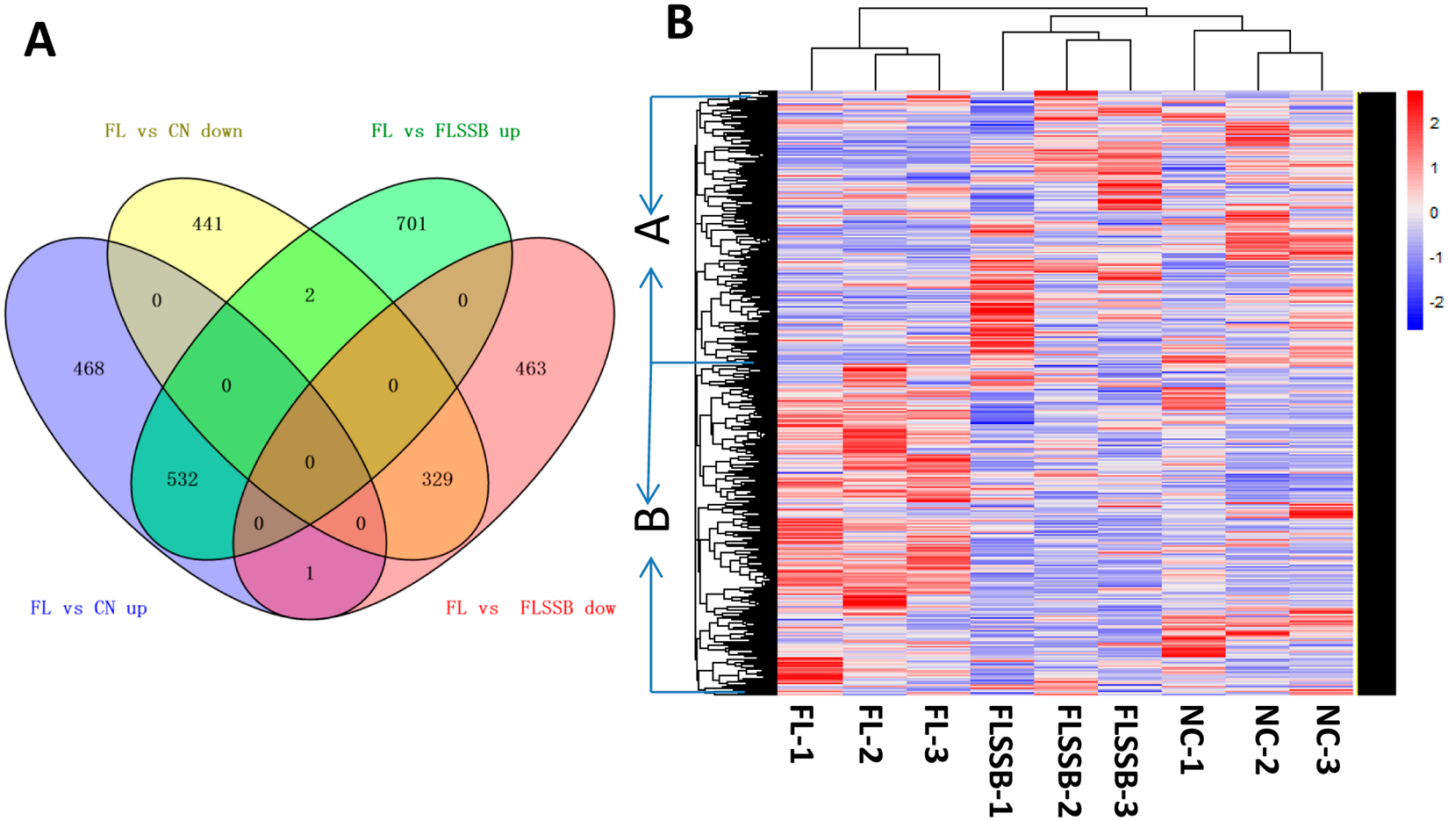
Gene expression patterns in NC, FL, and FLSSB groups. (**A**) Venn Diagram of DEGs between NC, FL, and FLSSB groups. (**B**) Heat map of gene expression patterns in NC, FL, and FLSSB groups.

In total, 1,773 genes showed significant differences in expression between the FL and NC samples: 1,001 showed up-regulation and 772 showed down-regulation in the FL sample compared to the NC sample. In the comparison of FLSSB and FL samples, 2,028 genes were identified as DEGs, including 1,235 that were up-regulated and 793 that were down-regulated in the FLSSB sample. Among these DEGs, 532 genes were down-regulated and 329 were up-regulated in both NC and FLSSB groups compared to the FL group (Fig. 5A). A few genes showed inverse changes between the NC and FLSSB groups. To characterize patterns of differential gene expression in response to SSB extract treatment, a heat map was constructed to visualize the expression of the DEGs. As shown in Fig. 5, DEGs were sorted into three expression cluster groups (Clusters FL, FLSSB, and NC; Fig. 5B), based on the similarity of their expression profiles. The expression pattern of the FLSSB group was similar to that of the NC group, and was largely separate from the FL group. Furthermore, global genes could be divided into two clusters (Clusters A and B): cluster A genes were mainly up-regulated in FLSSB and NC groups, and down-regulated in FL group; cluster B genes were primarily down-regulated in FLSSB and NC groups, while up-regulated in the FL group.

### Functional analysis of DEGs

**Figure 6:**
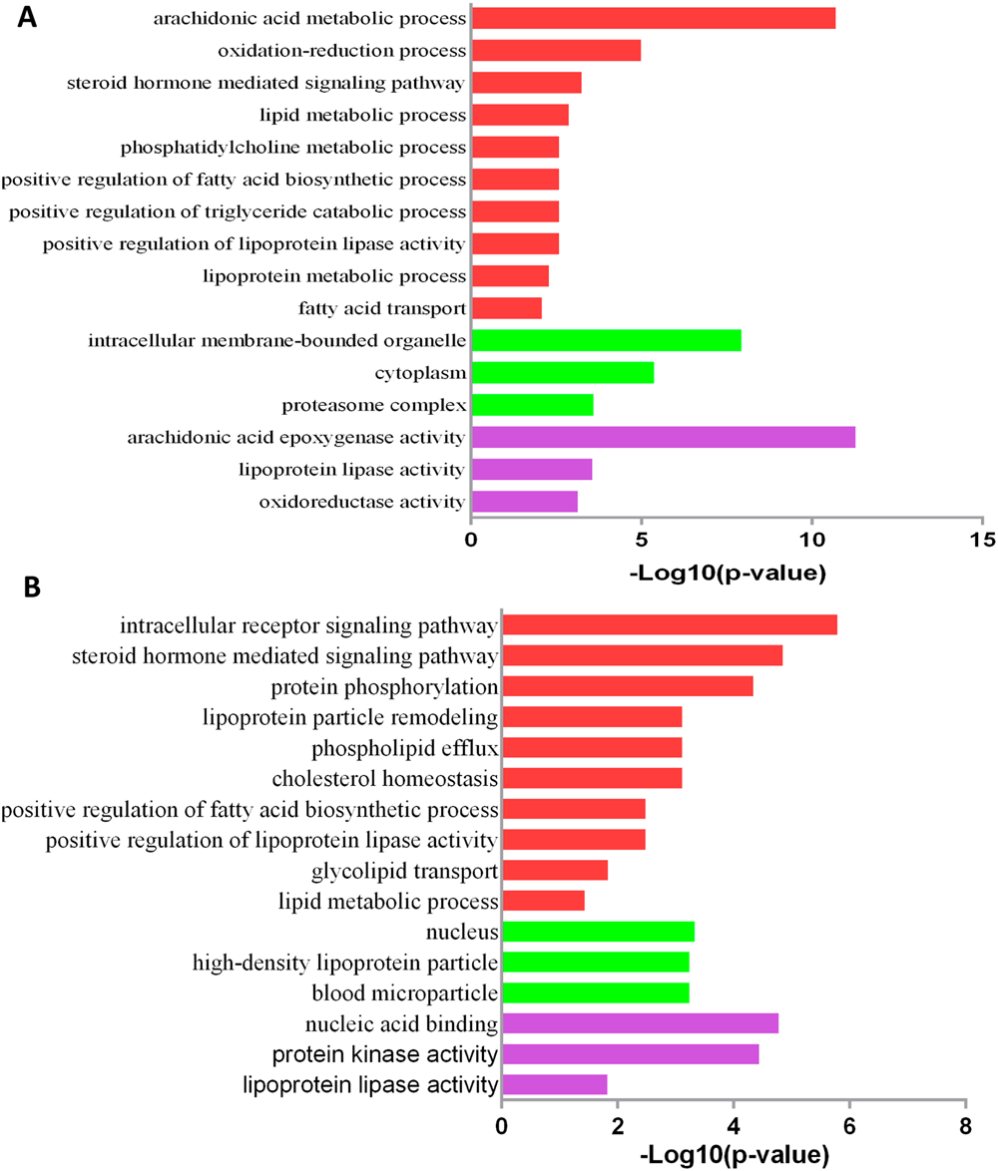
Biological processes analyses of DEGs. (**A**) GO analysis of DEGs between FL and NC groups. (**B**) GO analysis of DEGs between FL and FLSSB groups.

DEGs between FL and NC were assigned to 60 biological processes, 23 cellular component, and 54 molecular functions. For biological processes, oxidation-reduction process, lipid metabolic process, lipoprotein lipase activity, and fatty acid biosynthetic process were differently regulated between FL and NC (Fig. 6A); lipid metabolic process and metabolic process were mainly down-regulated in the FL group, while oxidation-reduction process and arachidonic acid metabolic process were mainly up-regulated. For DEGs between FLSSB and FL, 132 GO terms were significantly enriched, including 73 biological processes, 19 cellular component, and 40 molecular functions. The enriched biological processes were mainly associated with cholesterol homeostasis, lipoprotein particle remodeling, positive regulation of lipoprotein lipase activity, positive regulation of fatty acid biosynthetic process, glycolipid transport, and lipid metabolic process (Fig. 6B). Lipid metabolic process, metabolic process, protein dephosphorylation, and oxidation- reduction process were mainly up-regulated in the FLSSB group, while intracellular receptor signaling pathway, steroid hormone mediated signaling pathway, protein phosphorylation, lipoprotein particle assembly, and remodeling were mainly down-regulated.

### KEGG pathway analysis of DEGs

**Figure 7:**
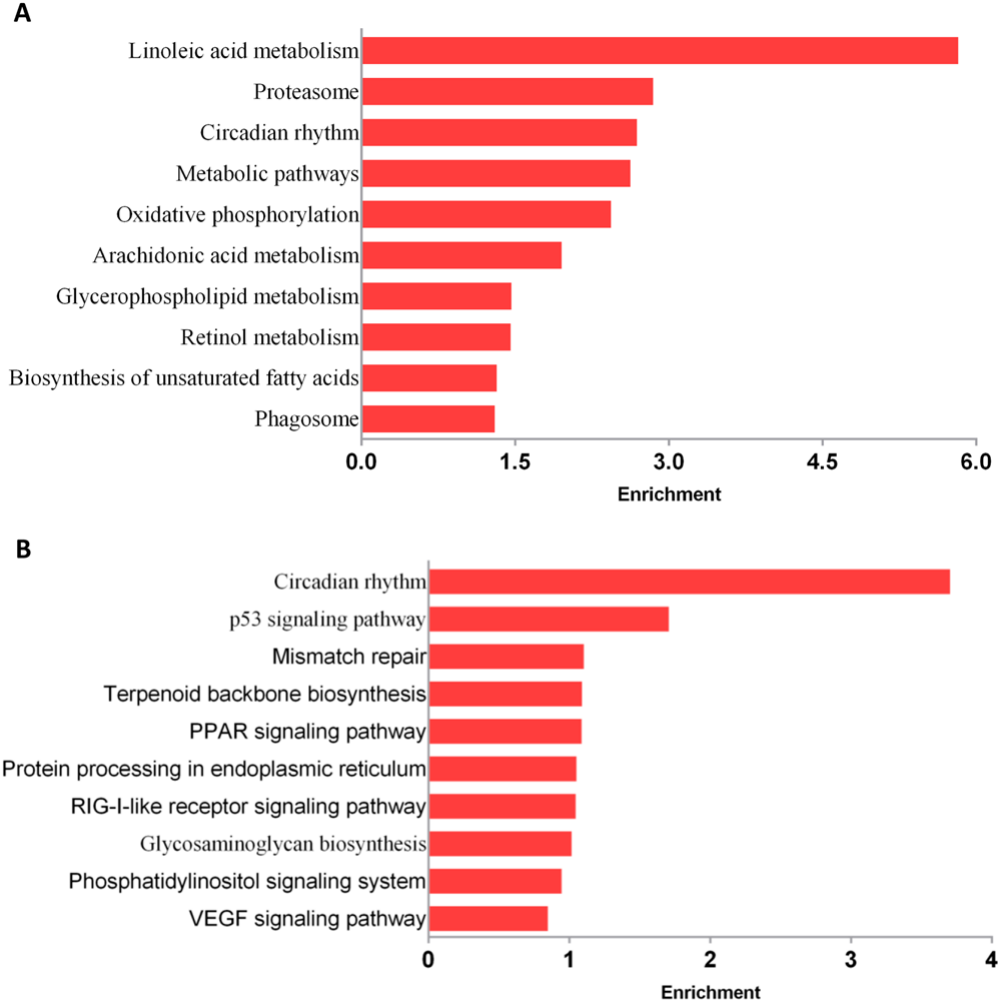
KEGG analysis of DEGs. (**A**) KEGG analysis of DEGs between FL and NC groups. (**B**) KEGG analysis of DEGs between FLSSB and FL groups.

A KEGG pathway analysis was performed for DEGs between the FL and NC groups to identify molecular mechanisms potentially involved in the development of fatty liver disease. Changes were found for metabolic pathways, oxidative phosphorylation, and phagosome (Fig. 7A). Genes involved in oxidative phosphorylation, metabolic pathways, and PPAR signaling were up-regulated in the FL group compared to the NC group; genes involved in linoleic acid metabolism, ABC transporters, and the p53 signaling pathway were mainly down-regulated. To explore the effect of SSB extract treatment, the pathways enriched for DEGs between FLSSB and FL were investigated. We found the SSB extract treatment altered pathways related to p53 signaling, PPAR signaling, and RIG-I-like receptor signaling (Fig. 7B).

### Dysregulated genes involved in p53 and PPAR signaling pathways

The KEGG analysis indicated that the p53 and PPAR signaling pathways were involved in the pathological process of fatty liver development, and that the SBB extract treatment significantly restored these pathways. With respect to genes in the PPAR signaling pathway, we found that lipoprotein lipase (*lpl*), long-chain fatty acid-CoA ligase 3 (*ACSL3*), acyl-CoA synthetase bubblegum family member 2 (*ACSBG2*), and long-chain fatty acid-CoA ligase 1 (*ACSL1*) were down-regulated in the FL group compared to the NC group. In the FLSSB group, *lpl* and *ACSL3* were up-regulated, and the retinoid X receptor (*RXR*), peroxisome proliferator activated receptor alpha (*PPARα*), peroxisome proliferator activated receptor delta (*PPARδ*) were down-regulated compared to the FL group. With regard to genes in the p53 signaling pathway, *Gadd45*, cyclin D, *fam237a*, cyclin G, and *Gadd45* were up-regulated, while *casp8* and cytochrome c were down-regulated in the FL group compared to the NC group. Moreover, *casp8*, brain-specific angiogenesis inhibitor 1 (*BAI1*), cytochrome c, cyclin D, and cytochrome c-b were up-regulated in the FLSSB group compared to the FL group, while cyclin D, cyclin G, and cyclin B2 were down-regulated.

**Figure 8:**
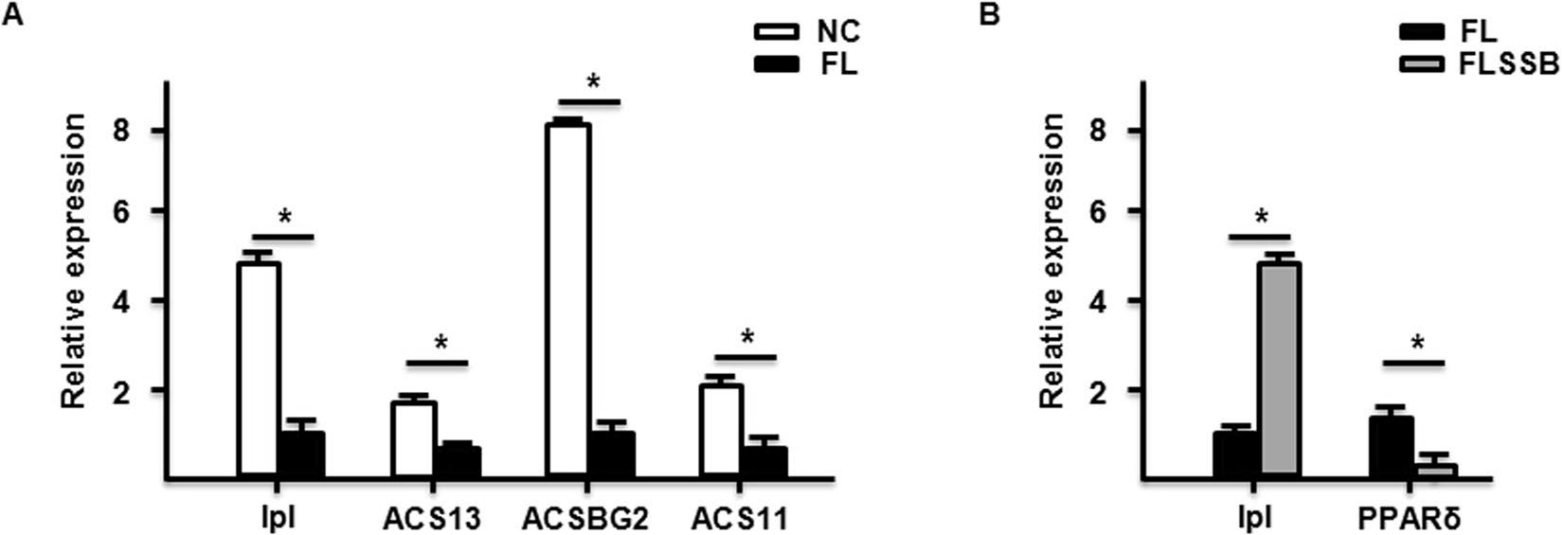
Verification of the selected DEGs. The expression of genes in the PPAR signaling pathway was analyzed by qRT-PCR in NC, FL, and FLSSB groups. A minimum of three independent experiments was performed. *P < 0.05, (t-test, n = 15).

In order to validate the sequencing results, some DEGs in the PPAR signaling pathway were selected for qRT-PCR analysis (Fig. 8). The results of the qRT-PCR analyses were consistent with those from the RNA-seq investigation (Fig. 8).

## Discussion

Recently, fatty liver disease has increased in frequency among cultured carp, grass-fish, and tilapia and is now of serious concern to the aquaculture industry. To investigate whether treatment of fish with an SSB extract might impede development of fatty liver disease, we first established a tilapia model of the disease. We confirmed this model using a combination biochemical index, histopathology, and hepatocyte apoptosis. The potential therapeutic effects of the SSB extract treatment were then explored and potential mechanisms of fatty liver disease investigated by RNA-seq.

The condition of the fish livers in the three groups was assessed using the grading system proposed by Brunt *et al*. (see “Materials and Methods”)^13^. In this study, cavitation affected 65% of the hepatocytes in the FL group, indicating that fatty liver disease had developed. Using Kuwashiro *et al*. ’s system for assessing non-alcoholic fatty livers, hepatocytes in the FL group were classified as severely diffuse, indicating the presence of severe fatty liver disease^6^. By contrast, the FLSSB group only developed a moderate fatty liver, suggesting that SSB extract treatment suppressed the development of fatty liver.

Biochemical markers can effectively reflect the hepatic function: an increase in LDH indicates hepatocyte injury^14^, and an increase in TG and CHOL are important blood lipid characteristics of fatty liver disease^15^. TBA is a cholesterol metabolite in the liver, and hepatic cell disease can induce an increase in TBA in the serum^16^. In the present study, serum levels of LDH, TG, and CHOL were significantly higher in the FL group compared to the NC and FLSSB groups. This suggested that SSB extract treatment improved liver function parameters, leading to improvements in serum levels. These results are consistent with a report that SSB extract treatment prevents fulminant hepatic failure in mice, and that this protection may be related to the anti-apoptotic activity of the extract and suppression of mitogen activated protein kinase activity^11^. Furthermore, since oxidative stress plays an important role in hepatic toxicity caused by diethylamine, the use of treatments with antioxidant activities will provide better protection against liver damage^17^. CAT, SOD, T-AOC, and GSH-Px are generally used as markers of antioxidant function^18^. MDA is a lipid peroxide formed by the oxygen free radical activities in cells and can interact with normal proteins and DNA to cause toxic and carcinogenic effects. In this study, we found that treatment with SSB extract increased the serum level of CAT, SOD, T-AOC, and GSH-Px and reduced MDA, suggesting that it might have a protective effect on the liver by reducing oxidative stress damage. Similarly, treatment of hamsters with wedelolactone, a plant-derived compound, was found to improve hepatic steatosis by increasing the SOD and GSH-Px activities and decreasing the level of MDA^19^. ALP levels are also of value for diagnosing and monitoring treatment of diseases associated with liver^20^, and TP is similarly of value for diseases related to the liver^21^. ALB transports essential fatty acids from adipose tissue, and reduction of ALB levels may be related to liver disease^22^. In the present study, treatment with the SSB extract decreased serum WBC, TP, and ALB levels. ALP, TP, GLOB, and ALB are used as biochemical markers for liver condition^23–25^. Development of a fatty liver caused reduction of ALP, TP, GLOB, and ALB levels. Treatment with SSB extract significantly increased ALP, TP, GLOB, and ALB levels suggesting that this treatment might improve fatty liver disease in the fish.

We identified a large number of DEGs between the FLSSB and FL groups. The KEGG pathway analysis revealed that p53 and PPAR signaling pathways might be involved in the pathogenesis of fatty liver development and in the recovery induced by SSB extract treatment. The PPARα signaling pathway has been reported to be involved in pathological processes of fatty liver development in rats with pancreatitis^26^. PPARα is a transcription factor and an important regulator of lipid metabolism in the liver. Activation of PPARα facilitates catabolism of fatty acids via upregulation of genes related to peroxisomal and mitochondrial fatty acid β-oxidation, fatty acid transport, fatty acid binding, and activation^27^. PPARα is mainly activated by ligand binding, which requires heterodimer formation with RXR^28^. Another member of the PPAR family, PPARδ, is a nuclear hormone receptor that may be related to the development of chronic diseases, including diabetes, obesity, atherosclerosis, and cancer^29,30^. Knockout studies in mice revealed the PPARδ plays an important role in epidermal cell proliferation, glucose metabolism, and lipid metabolism^31^. In keratinocytes, PPARδ stimulates cell differentiation and lipid accumulation^32^. PPARδ can also stimulate fatty acid accumulation in the livers of mice and possibly humans^33^. Garbacz *et al*. showed that PPARα downstream signaling is required for PPARδ agonist-induced weight loss and liver steatosis^34^. In the present study, expression of *PPARα*, *PPARδ* and *RXR* was significantly reduced in the FLSSB group compared to the FL group. Moreover, the PPAR target genes *lpl*, *ACSL3*, *ACSBG2*, and *ACSL1* were down-regulated in the FL group compared with the NC group, while SSB extract treatment increased expression of *lpl* and *ACSL3*. In *Sparus aurata*, bisphenol A1-contaminated diets induce lipid accumulation by upregulation of the levels of *PPARα* and *PPARδ* mRNAs and significantly downregulating *lpl* mRNA^35^. Wedelolactone treatment was found to regulate lipid metabolism and improve hepatic steatosis by enhancing the expression of *lpl*^11^. Overall, our findings indicate that SSB extract treatment may alleviate the fatty liver condition in fish, at least in part, through the PPAR signaling pathway.

In addition to the PPAR signaling pathway, several genes involved in the p53 pathway were also changed after SSB extract treatment. The p53 pathway governs a variety of biological processes, including DNA repair, cell cycle, and apoptosis initiation. Inhibition of p53 can reduce hepatic triglyceride accumulation and lipotoxicity in a mouse model of non-alcoholic fatty liver disease, by reducing malonyl CoA and favoring the β-oxidation of fatty acids^36^.

In conclusion, SSB extract treatment alleviated nonalcoholic steatohepatitis through a variety of mechanisms in tilapia. Our data showed the SSB extract treatment can restore the feed coefficient, immune capacity, and pathological index in fish with fatty liver, and prevent apoptosis of hepatocytes. We suggest that disruption of the p53 and PPARα signaling pathways may be involved in steatohepatitis development and that the therapeutic effect of SSB extract treatment is also mediated though these pathways. These findings shed new light on possible treatments of steatohepatitis.

## Material and Methods

### Animal studies

All protocols involving the fish were approved by the Animal Welfare Committee of Guangxi University and conformed to the procedures described in the Guide for the Care and Use of Laboratory Animals. SSB extract (Batch No: XR-160409) was obtained from Shaanxi Xinrui Biotechnology Co. (Xian, China). Similarly-sized tilapia were obtained from Guangxi Wuming Aquaculture Breeding Center (Wuming, China). After acclimatization in the laboratory for one week, 540 healthy fish were randomly divided into three groups: the normal control group were supplied with the basic feed (NC group); the non-alcoholic fatty liver disease model group were fed with a high-fat diet (FL group); and the experimental group were fed with the high-fat diet supplemented with 1.2 g/kg^−1^ SSB extract (FLSSB group). Each group was set up in triplicate. The fish were fed twice a day, at 9 am and 6 pm, at a daily feeding rate of 4% of body weight. The compositions of the three diets are shown in Table S1. The water in the fish tanks was maintained at 28.2 ± 1.3 °C, pH 7.2 ± 0.1, and dissolved oxygen content of 6.51 ± 0.43 mg/L. Treatments were continued for six weeks; the body weights of 15 randomly caught fish from each group were recorded each week. On the 42nd day, feeding was stopped for 24 h. The fish were weighed and weight gain, specific growth rate, and feed coefficient were calculated. Twelve fish were randomly selected from each group and euthanized using MS-222; the liver of each fish was dissected and weighed.

### Biochemical and enzyme analyses

Fifteen fish were randomly selected from each group. Approximately 4–5 ml blood per fish was extracted from the tail vein using a 1 ml syringe; the coagulated blood was centrifuged at 1000 × g at 4 °C for 10 min to collect the serum. Serum and visceral tissue were used for measurement of the levels of triglycerides (TG), aspartate transaminase (AST), alanine transaminase (ALT), and lactate dehydrogenase (LDH), and to determine the immunological markers cholesterol (CHOL), total bile acid (TBA), total protein (TP), albumin (ALB), globulin (GLOB), and alkaline phosphatase (ALP). These measurements were made using a 7600–120 automatic biochemical analyzer (Hitachi). The antioxidant markers catalase (CAT), superoxide dismutase (SOD), glutathione peroxidase (GSH-Px), total antioxidant capacity (T-AOC), and malondialdehyde (MDA) were analyzed using an Antioxidant Assay Kit (Jiangsu Zhiyu Biotechnology Co. LTD, Jiangsu, China). The levels of protease, lipase and amylase in hepaticpancreas and intestine were measured using a Digestive Enzyme Assay Kit (Jiangsu Zhiyu Biotechnology Co. LTD).

### Liver biopsy examination

Liver histological sections were obtained from three fish in each group. The sections (5 µm) were stained with hematoxylin and eosin (H&E). Fatty liver degeneration was assessed using the established medaka fish non-alcoholic fatty liver model scoring system: slight (1 point), development of fat is limited to the area surrounding blood vessels; moderate (2 points), mild to severe fat development; severe (3 points), fat degeneration with diffuse hepatocytes. Assessment of hepatocytes (nuclear) was performed by light microscopy (×400 lens): cells are ordered and nucleus is clear (0 point); the nucleus was moved on to the edge (1 point); small degree of nuclear rupture and disintegration (2 points); most nuclei show rupture, hepatocytes are disordered, and necrotic lesions are present (3 points).

Hepatic cell vacuolar degeneration was scored as follows: no vacuolar degeneration (0 point); 20% degeneration (1 point); 20–40% degeneration (2 points); >40% degeneration (3 points). In this experiment, the liver disease model is considered to be successfully established when the average scores of the above three methods are greater than 2 points.

### TUNEL assay

Apoptosis in hepatocytes was assessed using the terminal deoxynucleotidyl transferase (TdT)-mediated dUTP nick end labeling (TUNEL) assay following the manufacturer’s instructions (Dead End™ Fluorometric TUNEL System, Promega, Madison, WI, USA). After permeabilization with 0.2% Triton X-100, cells were labeled with fluorescein isothiocyanate-12-dUTP and stained with 1 μg/ml propidium iodide. Signal detection was performed at 520 ± 20 nm under a fluorescence microscope (EVOS™fl Digital Inverted Fluorescence Microscope, Fisher Scientific, Paisley, Scotland, UK).

### Total RNA isolation and sequencing

Total RNA was isolated using TRIzol reagent (Invitrogen, Carlsbad, CA, USA), purified using an RNeasy mini kit (Qiagen, Benelux BV, Venlo, Netherlands) according to the manufacturer’s instructions, and quantified using a NanoDrop 1000 spectrophotometer v3.7 (NanoDrop Technologies, Inc., Wilmington, DE, USA). cDNA synthesis, library preparation (200-bp inserts), and Illumina sequencing (90-bp paired-end reads) were performed at Shanghai Yinbio Technology Co. LTD (Shanghai, China).

### Transcript assembly and differential expression analysis

Raw reads were cleaned to obtain high-quality data for analysis, including removal of reads and adapters. The obtained clean reads were then mapped to the *Oreochromis niloticus* (Nile tilapia) genome using TopHat version v2.0.12^37^. The expression profile was estimated using the Cufflinks package^38^. The transcripts were reconstructed using Cufflinks based on genome annotation, and then merged by cuffmerge. Expectation-maximization (RSEM) was used to analyze gene expression. Transcript abundance for a given gene was quantified by calculating the frequency of aligned RNA-seq reads, and this was expressed as an RPKM value (reads per kilobase of transcript sequence per million mapped reads). After normalization of the read count using the trimmed mean of M-values (TMM)^39^, differentially expressed genes (DEGs) were identified between two samples using DEGseq^40^. The threshold of the p-value was determined by the false discovery rate (FDR), and the threshold for significant differential expression was set at FDR < 0.05 and | log2 (fold change) | >1.

### Gene ontology (GO) and Kyoto Encyclopedia of Genes and Genomes (KEGG) analysis

Enrichment analysis using GO and KEGG terms were applied to determine the roles of DEGs in different GO terms or biological pathways, using DAVID software packages (https://david.ncifcrf.gov/summary.jsp). Hierarchical clustering was performed to distinguish gene expression profiles among samples.

### Quantitative reverse transcription-polymerase chain reaction (qRT-PCR)

Total RNA was extracted with TRIzol reagent. First-strand cDNA was synthesized using the RT2 First Strand Kit or NCode TM miRNA First-Strand cDNA Synthesis Kit (Life Technologies). qRT-PCR was performed in an ABI Q6 detection system (Applied Biosystems, Foster City, CA, USA) using a Real Time SYBR master mix kit. qRT-PCR was performed under the following conditions: 95 °C for 10 min; 95 °C for 15 s, and 60 °C for 1 min (40 cycles). The relative expression levels of *lpl*, *ACS13*, *ACSBG2*, *ACS11*, and *PPAR*δ were calculated with the 2^−ΔΔCt^ method, with normalization to β-actin mRNA.

### Statistical analysis

Statistical differences among the three groups were calculated by one-way analyses of variance (ANOVA) using SPSS software (Statistical Package for Social Sciences, SPSS Corporation, Chicago, USA). Statistical differences between two groups were compared by *t*-tests. All values are expressed as the mean ± standard deviation (SD) and P < 0.05 was considered statistically significant.

## Electronic supplementary material

Nutrient level and composition of experimental diet (%).

Effects of Sedum sarmentosum Bunge on Antioxidant index of Tilapia.

Effects of Sedum sarmentosum Bunge on the liver of Tilapia.

Effects of Sedum sarmentosum Bunge on the digestive enzyme of Tilapia.

The percentage of vacuoles in liver slices of three groups after 6 weeks.

Pathological index score statistics of three groups after 6 weeks.

Statistics of data production.

Statistics of Mapping.
