# Supplementary material for: Ameliorative effect of Sedum sarmentosum Bunge extract on Tilapia fatty liver via the PPAR and P53 signaling pathway
Source: Sci Rep. 2018 May 31;8:8456. doi: 10.1038/s41598-018-26084-2 (PMC5981579; doi:10.1038/s41598-018-26084-2)
Supplement: Supplementary file 1 — Nutrient level and composition of experimental diet (%). [file 41598_2018_26084_MOESM1_ESM.pdf]

# Ameliorative effect of *Sedum sarmentosum* Bunge extract on Tilapia fatty liver via the PPAR and P53 signaling pathway

Lida Huang[1,2&], Yuan Cheng[1,3&], Kai Huang[1*], Yu Zhou[3*], Yanqun Ma[1], Mengci Zhang[1]

[1] College of Animal Science and Technology of Guangxi University, Nanning, China

[2] Zhanjiang Haiyuan Biological Technology Co. Ltd.

[3] Guangxi Academy of Fishery Sciences, Nanning, China

[&]Equal contributors

*Correspondence and requests for materials should be addressed to K.H. (email: kaihuangnn1@163.com) or Y.Z. (email: zy123000@qq.com)

**Supplementary Table S1**: Nutrient level and composition of experimental diet(%).

| Material | NC | FL | FLSSB |
|---|---|---|---|
| Fish meal | 3 | 3 | 3 |
| Soybean meal | 35 | 35 | 35 |
| Rape meal | 22 | 22 | 22 |
| Peanut meal | 10 | 10 | 10 |
| Rice bran | 8 | 8 | 8 |
| Corn | 18.2 | 13.95 | 13.83 |
| Calcium phosphate | 1 | 1 | 1 |
| Choline Choride | 1.5 | 1.5 | 1.5 |
| Vitamins premix | 0.3 | 0.3 | 0.3 |
| Mineral premix | 0.5 | 0.5 | 0.5 |
| NaCl | 0.5 | 0.5 | 0.5 |
| Soybean oil | 0 | 4.25 | 4.25 |
| Sedum sarmentosum Bunge | 0 | 0 | 0.12 |
| Total | 100 | 100 | 100 |
