# Supplementary material for: Ameliorative effect of Sedum sarmentosum Bunge extract on Tilapia fatty liver via the PPAR and P53 signaling pathway
Source: Sci Rep. 2018 May 31;8:8456. doi: 10.1038/s41598-018-26084-2 (PMC5981579; doi:10.1038/s41598-018-26084-2)
Supplement: Supplementary file 2 — Effects of Sedum sarmentosum Bunge on Antioxidant index of Tilapia. [file 41598_2018_26084_MOESM2_ESM.pdf]

Ameliorative effect of *Sedum sarmentosum* Bunge extract on Tilapia fatty liver via the PPAR and P53 signaling pathway

Lida Huang[1,2&], Yuan Cheng[1,3&], Kai Huang[1*], Yu Zhou[3*], Yanqun Ma[1], Mengci Zhang[1]

[1]College of Animal Science and Technology of Guangxi University, Nanning, China

[2]Zhanjiang Haiyuan Biological Technology Co. Ltd.

[3]Guangxi Academy of Fishery Sciences, Nanning, China

[&]Equal contributors

*Correspondence and requests for materials should be addressed to K.H. (email: kaihuangnn1@163.com) or Y.Z. (email: zy123000@qq.com)

Supplementary Table S2: Effects of *Sedum sarmentosum* Bunge on Antioxidant index of Tilapia

Parameters	Groups		
	NC	FL	FLSSB
CAT (U/ml)	62.82±3.89[a]	36.04±2.07[c]	51.15±3.36[b]
SOD (U/ml)	172.41±1.76[a]	128.41±6.0	153.58±14.20
GSH-Px (U/L)	155.19±5.07[a]	98.63±4.58[c]	141.20±4.29[b]
MDA	9.36±0.55[c]	13.19±0.44[a]	10.55±1.10[b]
T-AOC (U/ml)	26.85±1.14[a]	21.00±0.60[c]	22.90±0.27[b]

Values are means of 15 fishes from each group.

Values with different small superscript letter within same row indicate significantly different (ANOVA $P < 0.05$).
