# Supplementary material for: Ameliorative effect of Sedum sarmentosum Bunge extract on Tilapia fatty liver via the PPAR and P53 signaling pathway
Source: Sci Rep. 2018 May 31;8:8456. doi: 10.1038/s41598-018-26084-2 (PMC5981579; doi:10.1038/s41598-018-26084-2)
Supplement: Supplementary file 3 — Effects of Sedum sarmentosum Bunge on the liver of Tilapia. [file 41598_2018_26084_MOESM3_ESM.pdf]

Ameliorative effect of *Sedum sarmentosum* Bunge extract on Tilapia fatty liver via the PPAR and P53 signaling pathway

Lida Huang[1,2&], Yuan Cheng[1,3&], Kai Huang[1*], Yu Zhou[3*], Yanqun Ma[1], Mengci Zhang[1]

[1]College of Animal Science and Technology of Guangxi University, Nanning, China

[2]Zhanjiang Haiyuan Biological Technology Co. Ltd.

[3]Guangxi Academy of Fishery Sciences, Nanning, China

[&]Equal contributors

*Correspondence and requests for materials should be addressed to K.H. (email: kaihuangnn1@163.com) or Y.Z. (email: zy123000@qq.com)

Supplementary Table S3: Effects of *Sedum sarmentosum* Bunge on the liver of Tilapia

Parameters	Groups		
	NC	FL	FLSSB
AST (U/L)	205.42±15.84^{c}	319.53±24.	255.87±16.24
ALT (U/L)	52.06±2.54^{c}	71.45±2.90^{a}	62.03±1.33^{b}
CAT (U/ml)	77.75±4.74^{a}	47.49±4.34^{b}	70.51±2.69^{a}
SOD (U/ml)	148.85±7.11^{a}	105.22±6.3	123.05±9.15 ^{b}
GSH-Px (U/L)	117.43±3.28^{a}	76.07±4.16^{b}	108.57±7.90^{a}
MDA	7.85±0.90^{c}	11.42±0.27^{a}	10.01±0.51^{b}
T-AOC (U/ml)	34.01±0.86^{a}	24.18±1.11^{c}	28.61±0.68^{b}

Values are means of 15 fishes from each group.

Values with different small superscript letter within same row indicate significantly different (ANOVA $P < 0.05$).
