# Supplementary material for: Ameliorative effect of Sedum sarmentosum Bunge extract on Tilapia fatty liver via the PPAR and P53 signaling pathway
Source: Sci Rep. 2018 May 31;8:8456. doi: 10.1038/s41598-018-26084-2 (PMC5981579; doi:10.1038/s41598-018-26084-2)
Supplement: Supplementary file 4 — Effects of Sedum sarmentosum Bunge on the digestive enzyme of Tilapia. [file 41598_2018_26084_MOESM4_ESM.pdf]

Ameliorative effect of *Sedum sarmentosum* Bunge extract on Tilapia fatty liver via the PPAR and P53 signaling pathway

Lida Huang[1,2&], Yuan Cheng[1,3&], Kai Huang[1*], Yu Zhou[3*], Yanqun Ma[1], Mengci Zhang[1]

[1]College of Animal Science and Technology of Guangxi University, Nanning, China

[2]Zhanjiang Haiyuan Biological Technology Co. Ltd.

[3]Guangxi Academy of Fishery Sciences, Nanning, China

[&]Equal contributors

*Correspondence and requests for materials should be addressed to K.H. (email: kaihuangnn1@163.com) or Y.Z. (email: zy123000@qq.com)

Supplementary Table S4: Effects of *Sedum sarmentosum* Bunge on the digestive enzyme of Tilapia

Parameters	Groups		
	NC	FL	FLSSB
Hepatopancreatic protease	1895.81±61.85^{c}	2718.99±35.2	2301.86±229.
Intestinal protease (U/ml)	1387.57±127.5	2352.56±83.8	1923.94±161.
Hepatopancreatic lipase	120.80±12.85^{b}	175.07±14.54^{a}	141.13±11.63
Intestinal lipase (U/L)	99.57±5.02^{c}	147.93±10.58^{a}	123.75±6.60^{b}
Hepatopancreatic amylase	37.30±2.19^{b}	49.18±2.53^{a}	40.80±0.94^{b}
Intestinal amylase (IU/L)	29.48±1.87^{c}	43.21±2.22^{a}	36.09±1.93^{b}

Values are means of 15 fishes from each group.

Values with different small superscript letter within same row indicate significantly different (ANOVA $P < 0.05$).
