# Supplementary material for: Ameliorative effect of Sedum sarmentosum Bunge extract on Tilapia fatty liver via the PPAR and P53 signaling pathway
Source: Sci Rep. 2018 May 31;8:8456. doi: 10.1038/s41598-018-26084-2 (PMC5981579; doi:10.1038/s41598-018-26084-2)
Supplement: Supplementary file 5 — The percentage of vacuoles in liver slices of three groups after 6 weeks. [file 41598_2018_26084_MOESM5_ESM.pdf]

Ameliorative effect of *Sedum sarmentosum* Bunge extract on Tilapia fatty liver via the PPAR and P53 signaling pathway

Lida Huang[1,2&], Yuan Cheng[1,3&], Kai Huang[1*], Yu Zhou[3*], Yanqun Ma[1], Mengci Zhang[1]

[1]College of Animal Science and Technology of Guangxi University, Nanning, China

[2]Zhanjiang Haiyuan Biological Technology Co. Ltd.

[3]Guangxi Academy of Fishery Sciences, Nanning, China

[&]Equal contributors

*Correspondence and requests for materials should be addressed to K.H. (email: kaihuangnn1@163.com) or Y.Z. (email: zy123000@qq.com)

Table S5 The percentage of vacuoles in liver slices of three group after 6 weeks

Groups	Percentage of vacuoles（%）
NC	0
FL	65
FLSSB	38
