# Supplementary material for: Ameliorative effect of Sedum sarmentosum Bunge extract on Tilapia fatty liver via the PPAR and P53 signaling pathway
Source: Sci Rep. 2018 May 31;8:8456. doi: 10.1038/s41598-018-26084-2 (PMC5981579; doi:10.1038/s41598-018-26084-2)
Supplement: Supplementary file 7 — Statistics of data production. [file 41598_2018_26084_MOESM7_ESM.pdf]

Ameliorative effect of *Sedum sarmentosum* Bunge extract on Tilapia fatty liver via the PPAR and P53 signaling pathway

Lida Huang[1,2&], Yuan Cheng[1,3&], Kai Huang[1*], Yu Zhou[3*], Yanqun Ma[1], Mengci Zhang[1]

[1]College of Animal Science and Technology of Guangxi University, Nanning, China

[2]Zhanjiang Haiyuan Biological Technology Co. Ltd.

[3]Guangxi Academy of Fishery Sciences, Nanning, China

[&]Equal contributors

*Correspondence and requests for materials should be addressed to K.H. (email: kaihuangnn1@163.com) or Y.Z. (email: zy123000@qq.com)

Supplementary Table S7: Statistics of data production.

SampleName	Encoding	TotalReads_Before	TotalBase_Before	TotalReads_After	TotalBase_After	Reads Filter %	Base Filter %	GC%_Before	GC%_After
A_1	Sanger / Illumina 1.9	34066378	5.11E+09	24707550	3.7E+09	0.725277	0.723681	47	47
A_2	Sanger / Illumina 1.9	41499260	6.22E+09	30798442	4.61E+09	0.742144	0.740916	47	46.5
A_3	Sanger / Illumina 1.9	40824004	6.12E+09	31181054	4.67E+09	0.763792	0.762384	47	47
B_1	Sanger / Illumina 1.9	41241326	6.19E+09	30933934	4.63E+09	0.750071	0.74881	47	47
B_2	Sanger / Illumina 1.9	37246186	5.59E+09	27164374	4.07E+09	0.72932	0.728052	46.5	46
B_3	Sanger / Illumina 1.9	40400702	6.06E+09	29337014	4.39E+09	0.726151	0.72492	48	48
C_1	Sanger / Illumina 1.9	41052484	6.16E+09	31012556	4.64E+09	0.755437	0.754286	47.5	47
C_2	Sanger / Illumina 1.9	34002828	5.1E+09	25684916	3.85E+09	0.755376	0.754222	47.5	47
C_3	Sanger / Illumina 1.9	36325652	5.45E+09	26589516	3.98E+09	0.731976	0.730649	48	47
