# Supplementary material for: Ameliorative effect of Sedum sarmentosum Bunge extract on Tilapia fatty liver via the PPAR and P53 signaling pathway
Source: Sci Rep. 2018 May 31;8:8456. doi: 10.1038/s41598-018-26084-2 (PMC5981579; doi:10.1038/s41598-018-26084-2)
Supplement: Supplementary file 8 — Statistics of Mapping. [file 41598_2018_26084_MOESM8_ESM.pdf]

Ameliorative effect of *Sedum sarmentosum* Bunge extract on Tilapia fatty liver via the PPAR and P53 signaling pathway

Lida Huang[1,2&], Yuan Cheng[1,3&], Kai Huang[1*], Yu Zhou[3*], Yanqun Ma[1], Mengci Zhang[1]

[1]College of Animal Science and Technology of Guangxi University, Nanning, China

[2]Zhanjiang Haiyuan Biological Technology Co. Ltd.

[3]Guangxi Academy of Fishery Sciences, Nanning, China

[&]Equal contributors

*Correspondence and requests for materials should be addressed to K.H. (email: kaihuangnn1@163.com) or Y.Z. (email: zy123000@qq.com)

Supplementary Table S8: Statistics of Mapping.

#Item	A_1	A_2	A_3	B_1	B_2	B_3	C_1	C_2	C_3
All	24707550	30798442	31181054	30933934	27164374	29337014	31012556	25684916	26589516
UnMapped	3486751	3966419	4378821	4823874	4407275	6178970	4345215	4159188	5073588
	0.141121	0.12878635	0.140432103	0.155941	0.162245	0.21062	0.140111	0.161931	0.190812
Mapped	21220799	26832023	26802233	26110060	22757099	23158044	26667341	21525728	21515928
MappedRate	0.859	0.871	0.86	0.844	0.838	0.789	0.86	0.838	0.809
UniqueMapped	19997024	25362773	25202389	24482322	21394822	21722003	25031422	20349982	20292570
UniqueMappedRate	0.809	0.824	0.808	0.791	0.788	0.74	0.807	0.792	0.763
RepeatMapped	1223775	1469250	1599844	1627738	1362277	1436041	1635919	1175746	1223358
JunctionAllMapped	10436684	13585903	13668448	12920219	10678649	10852780	13833194	10723926	10440220
JunctionUniqueMapped	9649874	12632494	12597512	11891897	9847849	9993494	12735802	9988133	9681448
AllBase	3.7E+09	4612129206	4668570141	4.63E+09	4.07E+09	4.39E+09	4.64E+09	3.85E+09	3.98E+09
UnMappedBase	5.22E+08	593866170	655540442	7.22E+08	6.6E+08	9.25E+08	6.51E+08	6.23E+08	7.59E+08
MappedBase	3.18E+09	4018263036	4013029699	3.91E+09	3.41E+09	3.47E+09	3.99E+09	3.22E+09	3.22E+09
UniqueMapp	2.99	37981	37734	3.67	3.2E	3.25	3.75	3.05	3.04

edBase	E+09	94434	28188	E+09	+09	E+09	E+09	E+09	E+09
RepeatMapp<br>edBase	1.83<br>E+08	22006<br>8602	23960<br>1511	2.44<br>E+08	2.04<br>E+08	2.15<br>E+08	2.45<br>E+08	1.76<br>E+08	1.83<br>E+08
